# Surface Functionalization and Targeting Strategies of Liposomes in Solid Tumor Therapy: A Review

**DOI:** 10.3390/ijms19010195

**Published:** 2018-01-09

**Authors:** Muhammad Kashif Riaz, Muhammad Adil Riaz, Xue Zhang, Congcong Lin, Ka Hong Wong, Xiaoyu Chen, Ge Zhang, Aiping Lu, Zhijun Yang

**Affiliations:** 1 School of Chinese Medicine, Hong Kong Baptist University, 7 Baptist University Road, Kowloon Tong, Kowloon, Hong Kong, China; kashif@life.hkbu.edu.hk (M.K.R.); zhangxueflora@163.com (X.Z.); cong_cong_lilly@126.com (C.L.); 16483081@life.hkbu.edu.hk (K.H.W.); cxyu2016@hkbu.edu.hk (X.C.); zhangge@hkbu.edu.hk (G.Z.); 2 School of Chemical and Biomolecular Engineering, The University of Sydney, Sydney, NSW 2006, Australia; mria8901@uni.sydney.edu.au

**Keywords:** liposomes, targeted drug delivery, solid tumor, targeting ligands, surface functionalization

## Abstract

Surface functionalization of liposomes can play a key role in overcoming the current limitations of nanocarriers to treat solid tumors, i.e., biological barriers and physiological factors. The phospholipid vesicles (liposomes) containing anticancer agents produce fewer side effects than non-liposomal anticancer formulations, and can effectively target the solid tumors. This article reviews information about the strategies for targeting of liposomes to solid tumors along with the possible targets in cancer cells, i.e., extracellular and intracellular targets and targets in tumor microenvironment or vasculature. Targeting ligands for functionalization of liposomes with relevant surface engineering techniques have been described. Stimuli strategies for enhanced delivery of anticancer agents at requisite location using stimuli-responsive functionalized liposomes have been discussed. Recent approaches for enhanced delivery of anticancer agents at tumor site with relevant surface functionalization techniques have been reviewed. Finally, current challenges of functionalized liposomes and future perspective of smart functionalized liposomes have been discussed.

## 1. Introduction

The complex biology of solid tumors with various biological barriers, e.g., mononuclear-phagocyte system uptake and extravasation through vascular-endothelial layer, and physiological factors, e.g., hypoxia, low pH and raised interstitial-fluid pressure, highlights the need to design and formulate an efficient delivery system for anticancer agents [1]. The clinical application of many anticancer drugs have been hindered due to their limited water solubility, pharmacokinetics and potential adverse effects [2]. It has been estimated that in the USA during 2017 about 0.6 million deaths may occur due to all forms of cancers [3]. As regards the estimated cancer deaths, lung cancer tops the list, followed by colorectal cancer, pancreatic cancer, breast cancer and prostate cancer [4]. It was estimated that about 50% of all cancer deaths in the UK were due to common cancers: lung, breast, bowel and prostate [5]. Chemotherapeutic drugs damage both normal and tumor cells [6,7,8,9]. The most affected cells are bone marrow, gonads (sex organs), gastrointestinal tract and skin (hair follicle cells), liver and kidneys [10]. 

Several nanocarriers including liposomes have been utilized for cancer therapy. However, other nanocarriers have certain limitations. Nanoparticles have limited loading capacity, <5% payload as compared to nanoparticle weight [11,12]. Another major issue with nanoparticles is burst release of payload before reaching the intended site [11]. Antibody or sensitive molecules are inactivated upon interaction with hydrogels [13]. Gold nanoparticles have issues, e.g., biodistribution, pharmacokinetics and cationic ligand toxicity [14,15]. Limitations of the nanocarriers need to be addressed before establishment of their role in cancer therapy. To decrease side effects and achieve the efficient treatment of cancer, targeted delivery of small quantity of anticancer drugs using liposomes as drug delivery system have been suggested [16]. Liposomes facilitates in intracellular-delivery of anticancer drugs and prolongs the retention-time of encapsulated payload in cancer cells [17]. Liposomes can play a vital role in resolving the issues i.e., off-target effects of anticancer drugs by improving the pharmacokinetic-profile and pharmacological properties [18,19]. 

Several strategies have been employed for delivery of anticancer agents at tumor site, e.g., active and passive targeting. Active targeting with a combination of other approaches, e.g., stimuli sensitivity, is the current approach in tumor therapy. In this connection, liposomes are grafted with a variety of targeting ligands, e.g., peptides, aptamers, antibody fragments, etc., using different surface engineering techniques. Surface functionalization of liposomes with a variety of approaches has played an important role in formulation of a liposomal delivery system for efficient targeting, endocytosis and therapeutic-response. Surface functionalization and modification of liposomes using a variety of techniques have aided in addressing the current limitations of liposomes. In this regard, theranostic-liposomes with simultaneous functionalities of diagnosis and treatment in a single formulation is the latest approach in nanomedicine [20]. Liposomes can be engineered for a specific application using suitable surface functionalization and modification techniques, providing the key benefits in cancer therapy of prolonged circulation time, enhanced cellular-uptake, higher payload accumulation at tumor site, by passing lysosomal degradation and stimuli responsive payload release at intended site.

This review article has been written with the following aims: To describe the current strategies for targeting of liposomes at malignant solid tumor sites, with a focus on latest trends in active targeting. Extracellular targeting, i.e., overexpressed receptors on cancer cells surface; intracellular targeting, i.e., over-expressed receptors in cytoplasm and nucleus; organelle targeting; and tumor-microenvironment or vasculature targeting have been reported. Targeting ligands for functionalization of liposomes with relevant surface engineering techniques have been described. Stimuli strategies for enhanced delivery of anticancer agents at requisite location using stimuli-responsive functionalized liposomes have been discussed. Recent approaches for overcoming the current limitations of liposomes and efficient delivery of anticancer agents at tumor site, e.g., functionalization of liposomes with two targeting ligands, modification of liposomes with simultaneous responsiveness to concurrent stimuli and dual functionalized liposomes, responsive to stimuli and grafted with targeting ligands have been reviewed. Further, the relevant functionalization techniques in designing and formulation of a smart liposome based nanocarrier have been explained. Finally, challenges and limitations of functionalized liposomes and future perspective of smart functionalized liposomes have been discussed.

## 2. Liposomes and Their Classification

Liposomes have onion-like structures, and are among the most extensively studied nanocarriers for delivery of anticancer agents to tumor site, using a single or combination of targeting strategies [21]. Liposomes are vesicles having one or more concentric phospholipid bilayers separated by aqueous compartments [22]. Liposomes were first reported by Alec Bangham (1921–2010) in a research article published in 1964. At first, vesicles were called as “Banghasomes” or “multilamellar smectic mesophases”, and then the word “liposomes” was proposed by Gerald Weissmann, in which “lipos” means fat and “soma” means body [23,24,25,26]. 

**Figure 1:**
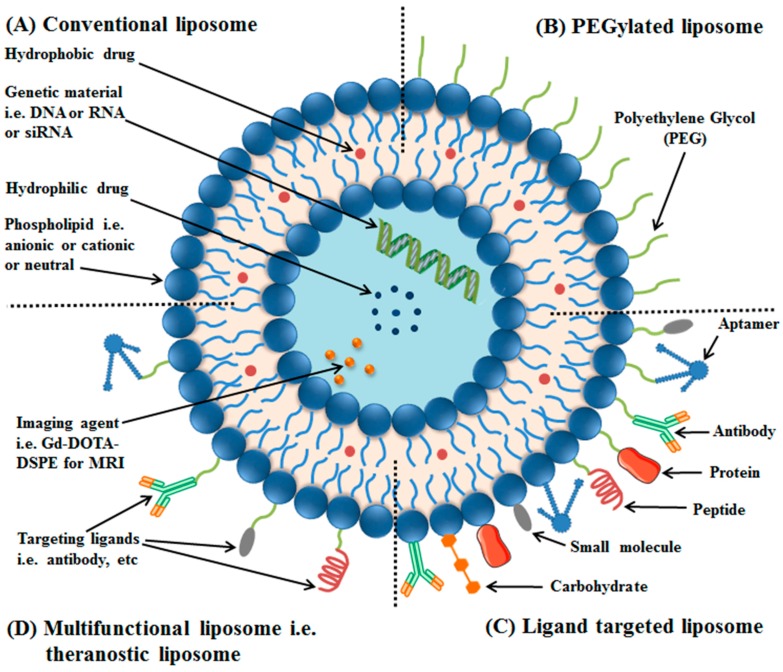
Liposomes: Conventional liposomes are made of phospholipids (**A**); PEGylated/stealth liposomes contain a layer of polyethylene glycol (PEG) at the surface of liposomes (**B**); targeted liposomes contain a specific targeting ligand to target a cancer site (**C**); and multifunctional such as theranostic liposomes, which can be used for diagnosis and treatment of solid tumors (**D**).

Conventional liposomes are made up of phospholipid bilayers, and, when administered intravenously, are taken up by the reticuloendothelial system (RES), thus have short circulation times. Opsonin (a serum-protein) recognizes them as foreign substance, therefore, they are destroyed by phagocytes (a part of RES). To increase the circulation time, liposomes surface has been coated with a hydrophilic polymer, i.e., polyethylene glycol (PEG), increasing repulsive forces between liposomes and serum-components. Such surface modified liposomes have been termed as PEGylated or stealth liposomes (see Figure 1) [27]. 

Functionalization of liposomes with a suitable ligand, i.e., peptides, antibodies or their fragments, aptamers, small molecules, etc., for targeted delivery of anticancer agents at the requisite site, i.e., tumor, using overexpressed receptors as a docking-site have resulted in formation of ligand-targeted liposomes or targeted liposomes. Application of targeted liposomes, formulated by a variety of surface engineering techniques in cancer therapy, has demonstrated minimum off-target effects to healthy tissues. Immunoliposomes have been formulated by chemically coupling antibodies or their fragments to the liposomal surface, resulting in high specificity for their target antigens [28].

Multifunctional liposomes have the potential to overcome the current challenges of liposomal formulations with a single functionality. A combination of surface functionalization and modification techniques has been utilized to formulate a nanoscale liposomal formulation, with a variety of functionalities. Liposomes having two ligands, e.g., two peptides (dual functional liposomes) [29], liposomes carrying two ligands and two anticancer drugs [30], liposomes with a targeting ligand and an imaging agent [31,32,33], theranostic liposomes having an imaging and therapeutic agent [34,35,36], etc., have been reported.

The applications of liposomes can be divided into two sections, i.e., application in cancer therapy and applications in other fields, e.g., fungal infection, gene therapy, as vaccine carriers, etc. [16,21,27,28,36,37,38,39]. Despite the large number of research publications regarding formulation and application of liposomes in cancer therapy, only few commercial anticancer liposomal formulations are available in the market [40,41]. 

## 3. Strategies for Targeting of Anticancer Agents at Requisite Site

The conventional approach for cancer therapy results in low accumulation of anticancer agents at the requisite tumor site with associated off-target effects. Consequently, several strategies have been developed and utilized for targeting and delivery of anticancer agents at requisite location to attain the optimal response in cancer therapy using liposomes. Passive targeting (with enhanced-permeability and retention effect) and active targeting are the main strategies for the delivery of anticancer agents at tumor site [42,43,44,45,46,47].

### 3.1. Enhanced Permeability and Retention (EPR) Effect and Its Application in Tumor Therapy

Conventional delivery systems in cancer therapy show a significant interaction with reticuloendothelial-system (RES). Passive targeting involves the formulation of a delivery system or nanocarrier that can avoid the elimination by body defense mechanisms, i.e., phagocytosis. One way to increase circulation time is to prepare PEGylated liposomes. This strategy is called passive targeting. Cancer cells have enhanced permeability or leakiness. Further, blocked lymphatic drainage also causes the accumulation of macromolecules. The targeting strategy is also termed as enhanced permeability and retention (EPR) effect [48,49]. 

The advantage of EPR effect can be maximized by careful consideration of liposome’s particle size. Liposomes having a particle size range between 40 and 200 nm have demonstrated higher extravasation. Recently, suggestions were made to improve the EPR effect for the enhanced delivery of anticancer agents to tumor sites. The suggestions mainly include the use of a stimuli (internal or external) to increase cancer cell permeability [50]. 

### 3.2. Active Targeting with Surface Engineered Liposomes, Functionalized with Targeting Ligands

The current strategy is to target the anticancer agents, i.e., drug, at the diseased tissue site, i.e., tumor tissues, with minimum deposition at the non-targeted tissues or with lesser off target effects [51]. This approach resulted in direct targeting of payload at the requisite site, and is termed active targeting or ligand based targeting. This involves the attachment of a targeting ligand to the surface of liposomes which when administered to the cancer bearing mice tracks and targets the receptors on the diseased cells. Such liposomes are called as ligand targeted liposomes or targeted liposomes [52]. 

Ligands for active targeting have been attached directly to the lipids or attached to the distal end of PEG chains [53]. The ligand-lipid-PEG conjugate micelles can be incorporated into preformed liposomes by post-insertion technique. In this approach, stealth liposomes carrying PEG at the surface are incubated with the micelles, resulting in formation of targeted liposomes. Another widely used approach based on traditional liposome formulation method is incorporation of the ligand, i.e., lipid-PEG-ligand conjugate, into the liposome formulation step [54,55].

It has been suggested that the cell penetrating peptide (CPP) assist delivery of anticancer agents via nanocarriers such as liposomes in three ways: (1) through covalent conjugation of the drug molecules with a CPP; (2) encapsulation of drugs into CPP attached nanocarriers, e.g., liposomes; and (3) by physical adsorption of drugs with CPPs via electrostatic complexation [56]. 

An important aspect in tailoring a targeted liposomal system is optimization of ligand’s density on the liposomes surface using the relevant surface engineering techniques. Issues such as aggregation may result from increasing ligands density beyond an optimum level [57]. Further, targeting ligands provides better internalization of liposomes in cancer cells [58]. Active targeting also has an advantage, i.e., decreased nonspecific distribution to undesired tissues [59]. Active targeting via liposomes possesses high specificity and targeting efficiency. The method of surface functionalization varies according to the final application of liposomal formulations (see Table 1) [60].

#### 3.2.1. Targeting of Over-Expressed Receptors on Cancer Cell’s Surface

**Figure 2:**
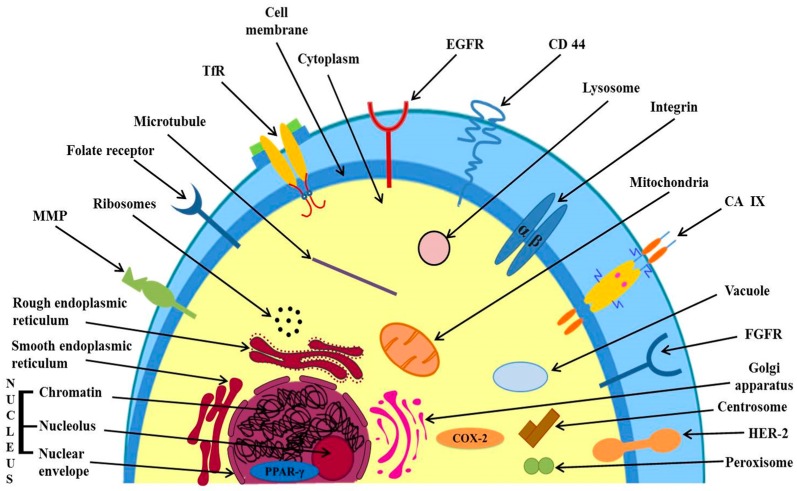
Targets (extracellular and intracellular receptors or over-expressed proteins) for active drug targeting in cancer therapy. Targets may also be organelles, e.g., lysosomes and mitochondria.

Several receptors are over-expressed in cancer cells as compared to normal cells. Targeting the over expressed receptors is a vital approach and forms the basis of active targeting leading to higher uptake and accumulation of anticancer agents in cancer cells at the tumor site [71]. Figure 2 shows extracellular and intracellular targets for active targeting of anticancer agents in cancer therapy.

##### Targeting of Epidermal Growth Factor Receptor (EGFR)

EGFR, e.g., HER1, a protein, tyrosine-kinase receptors are surface receptors over-expressed in many solid tumors, such as colorectal cancer (CRC), non-small cell lung cancer (NSCLC), breast, ovary and prostate. EGFR family contains four receptors: EGFR or HER1, HER2, HER3 and HER4 [72,73]. Mamot et al. prepared doxorubicin containing liposomes which were composed of DSPE, cholesterol, and MPEG-DSPE FabV fragments of cetuximab. The FabV fragments were covalently linked to the outer end of DSPE-PEG-MAL chains. Incorporation of DSPE-PEG-MAL-FabV was done by co-incubation with preformed liposomes at 55 °C for 30 min. These EGFR directed immunoliposomes target EGFR. The results showed better internalization within cancer cells and regression in human breast cancer model (MDAMB-68) versus non-targeted liposomes [61]. 

Cationic liposomes containing small interfering RNA (siRNA) were developed to target EGFR by conjugation of thiolated antibody with the maleimide (MAL) group at distal end of DSPE-PEG-MAL chains of preformed liposomes (0.2:1, Ab and MAL molar ratio) by overnight incubation at 4 °C with mild shaking. The suppression of lung cancer metastasis in mouse was observed. The liposomes showed an efficient transfer of siRNA to mouse (transfection) compared to non-targeted liposomes [62]. Anti-EGFR immunoliposomes containing celecoxib for the treatment of cancer were prepared. It was suggested that this strategy appears to be promising for treatment of cancer that over-express EGFR [74]. 

Anti-HER2 liposomes containing doxorubicin showed an improved drug delivery and antitumor activity against over expressing HER2 cancer cells compared to non-targeted liposomes. The liposomal formulations produced marked therapeutic results in different HER2-overexpressing tumor xenograft models. Fragments of trastuzumab have been utilized as a ligand for targeting HER2 receptor. The liposomes were developed by covalent conjugation of Fab or scFv to drug loaded liposomes by thioether linkage of free thiol of Ab-fragment and MAL group. Alternatively, thiol group was conjugated to terminal MAL group present on surface of liposomes. Doxorubicin was loaded in preformed immunoliposomes by ammonium sulfate gradient [63]. HER2 test has a key significance that it can be used for the diagnosis of breast cancer [75].

##### Targeting of Fibroblast Growth Factor Receptors (FGFRs)

These are transmembrane tyrosine kinases. FGFRs family has four members: FGFR 1, FGFR 2, FGFR 3 and FGFR 4. These receptors bind with fibroblast growth factors (FGFs) and this binding activates intracellular signaling. The elevated levels of all four FGFRs are seen in many cancers such as prostate, bladder, lung, etc. [76,77]. In a study by Rusnati et al. [78], a significant interaction of liposome-encapsulated FGF-2 with FGFR was observed compared to free FGF 2 with FGFR.

##### Targeting of Folate Receptors (FRs)

Folate Receptors are surface receptors, over expressed on various cancer cells such as lung, breast, etc. [79]. In a study by Low et al. [79], it was found that FR targeted doxorubicin liposomes showed higher cytotoxicity than plain doxorubicin liposomes. Folate targeted liposones were prepared by conjugation of folate to the liposomes using a PEG spacer by incorporation of PEGylated lipid in liposomes.

##### Targeting of Transferrin Receptors (TfRs)

Transferrin (Tf) is a serum iron Fe^3+^ carrier protein with mol wt about 80kDa. TfRs (TfR1 and TfR2 or CD77) are receptor for transferrin (Tf) and are present on the cell surface. Tf-TfR complex is internalized by endocytosis [80]. Due to increased iron demand by cancer cells, transferrin receptors are over expressed in cancers. TfR-targeted doxorubicin liposomes showed an improved therapeutic activity against liver cancer [81]. An increased cytotoxicity of TfR targeted liposomes containing encapsulated docetaxel as compared to non-targeted docetaxel liposomes was observed [82]. 

#### 3.2.2. Targeting Directly to the Desired Organelle, Over-Expressed Receptors in Cytoplasm and Nucleus

##### Targeting Directly to the Desired Organelle

The delivery of anticancer agents directly to desired organelle within cytoplasm is more appropriate than non-specific delivery to the organelles. The studies have been made involving drug targeting at lysosomes and mitochondria.

(a) Mitochondrial targeting 

Liposomes with triphenylphosphonium (TPP) moiety as a ligand is effective in the transfer of drug load to mitochondria. Paclitaxel liposomes with TPP or rhodamine 123 targeting ligand showed mitochondrial targeting with increased cytotoxicity as compared to non targeted liposomes [68,69]. 

Recently, dual functional paclitaxel liposomes with pH response and mitochondrial targeting were reported. The liposomes were effective in treating A549 cancer cells and A549 drug-resistant cancer cells. It was concluded that dual functional liposomes may provide a new approach for treating multidrug resistant cancer [70].

(b) Lysosomal targeting

Ceramides induce permeability in lysosome membrane. The delivery of ceramides to lysosomes using transferrin modified liposomes showed increased cell death in cancer cells [83]. 

##### Targeting Over-Expressed Receptors in Cell Cytoplasm and Nucleus

The examples include Cyclooxygenase-2 (COX-2) in the cytosol and Peroxisome Proliferator Activated Receptor-γ (PPAR-γ) in the nucleus.

(a) Targeting of Peroxisome proliferator-activated receptor γ (PPAR-γ)

It is a nuclear receptor protein. In CRC cancer tissues, a high level of PPAR-γ mRNA was found [84]. It appears from the literature survey that probably no study has been made to target this receptor using an anticancer drug carrier. 

(b) argeting of Cyclooxygenase-2 (COX-2)

Prostaglandin H_2_ synthase or cyclooxygenase (COX) is an enzyme which catalyzes the conversion of arachidonic acid (AA) to prostaglandin (PG). PGs play a role in inflammation [85]. COX-2 is over-expressed in different cancers such as prostate, colorectal, lung, etc. [86]. For cancer therapy and prevention, a strategy may be inhibition of its pathway. Bertagnolli et al., (2009) reported the inhibition of colorectal adenoma by the use of celecoxib (a selective COX-2 inhibitor used in chronic inflammation) but also reported increased cardiovascular risks [87]. In a study by Mikhaylova et al., liposomal-mediated delivery of COX-2-specific siRNA was used to down regulate COX-2 pathways in MDA-MB-231 breast cancer cells [88].

#### 3.2.3. Tumor Microenvironments Targeting

**Figure 3:**
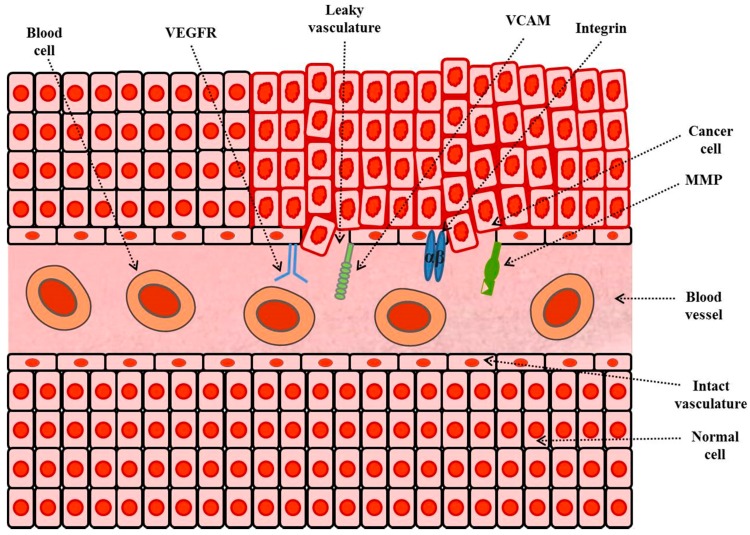
Targets (receptors or over-expressed proteins) in tumor microenvironment or vasculature for active drug targeting in cancer therapy.

Another approach is to target over-expressed receptors in tumor microenvironments/tumor vasculature/endothelium of tumor neovasculature such as tumor blood vessels. The formation of new blood vessels is essential to supply blood for tumor growth. The destruction of vasculature (arrangement of blood vessels in an organ) of a cancer diminishes the growth of cancer cells. A number of receptors are over-expressed in tumor microenvironment which can be targeted for efficient delivery of anticancer agents at the requisite site [89]. The examples of such targets include VEGFR, VCAMs (e.g., vascular cell-adhesion molecule 1 (VCAM 1)), MMPs such as membrane type-1-MMP (MT1-MMP) and αβ integrins. Figure 3 shows targets in tumor-microenvironment or vasculature for active drug targeting in cancer therapy.

##### Targeting of Vascular Cell-Adhesion Molecules (VCAMs)

These are involved in inflammation process. VCAM 1 is an antigen, which is over-expressed on NSCLC cells and cancer vessels [48,90]. In a study by Chiu et al., binding of the anti-VCAM-1 immunoliposomes was investigated. The binding of Ab-conjugated liposomes containing 2% DSPE-PEG(2000) was eight times higher than un-conjugated antibody liposomes [91]. Another study showed that VCAM 1 targeted liposomes accumulated in tumor blood vessels in mice having tumor xenograft [92].

##### Targeting of Integrins

These are transmembrane glycoproteins, over-expressed in neo-vascular epithelial tumor cells. A tripeptide RGD (Arg-Gly-Asp) has strong affinity for integrins. Liposomes coupled with RGD were prepared to target integrins. RGD liposomes having encapsulated paclitaxel showed an increased drug concentration in tumor cells than non-targeted paclitaxel liposomes [93]. Doxorubicin liposomes which were coupled with RGD showed a higher cellular uptake of the drug in the U87MG cell line compared with the simple doxorubicin liposomes [94].

##### Targeting of Matrix-Metalloproteases (MMPs)

MMPs are a family of enzymes (proteins) that can degrade extracellular matrix (ECM) and also play a role in the formation of new blood cells (angiogenesis) [95]. Several MMPs particularly MT1-MMP are expressed on tumor tissues. Anti-MT1-MMP Fab liposomes encapsulated with doxorubicin showed an enhanced cellular uptake in HT 1080 cancer cells (with over-expressed MT1-MMP) than non-targeted liposomes. The liposomes were formulated by conjugation of Fab fragments to the MAL moiety in preformed liposomes at a molar ratio of 1:3 in an incubation at 4 °C for 20 h [65]. A recent review on MMPs describes their role in cancer [96]. 

##### Targeting of Cluster-of-Differentiation 44 (CD44)

CD44 is a receptor protein (transmembrane adhesion molecule). It is over-expressed in many tumors such as colon, breast, etc. Since CD44 is a specific receptor for hyaluronic acid, hyaluronic acid modified mesoporous silica nano particles loaded with doxorubicin were prepared for targeting CD44. Higher cytotoxicity was observed in cancer cell than non-target silica particles [97]. In another study, anti-CD44 aptamer-1 containing liposomes were prepared by deprotected Apt1 conjugation to the terminal MAL group in preformed liposomes at a 0.5:1 molar ratio by overnight incubation at 4 °C. The high binding preference of aptamer-1 liposomes for CD44 expressing cancer cells was observed [98].

## 4. Targeting Ligands for Surface Functionalization of Liposomes in Tumor Therapy

Liposomes are functionalized with various targeting ligands with a single or a variety of surface engineering techniques. Ligands are mostly chemically attached to the liposomes through interaction of reactive-groups on liposomes surface and specific groups present in the ligand. Covalent or non-covalent incorporation of targeting ligands can be done to tailor a targeted liposomal system for cancer therapy. Liposomes are functionalized with various targeting ligands based on three kinds of reaction, i.e., formation of amide bond between carboxyl and amino groups; disulfide bond formation by reaction of pyridyldithiols and thiol group; and thioethers bonds formation by reaction of MAL and thiol group [99].

In post-coating method, preformed liposomes having reactive groups, e.g., MAL on surface have been conjugated with PEG derivatives [100]. PEG-lipid micelles have been used in another method for incorporation of PEG-lipid conjugates into the liposome membrane without disturbing liposomes [101]. A key approach for surface engineering of liposomes with an appropriate targeting ligand is based on conjugation reaction between ligand’s thiol group and MAL group on liposome’s surface.

Liposomes surface functionalization is commonly done with antibodies or their fragments, peptide (cell targeting or cell penetrating), aptamers and small molecules, e.g., folate, depending upon their application in tumor therapy. 

### 4.1. Surface Modification of Liposomes with Antibody

Antibodies or their fragments can be conjugated to the liposome’s surface to obtain immunoliposomes, using various surface engineering techniques. One approach for surface functionalization of liposomes is covalent linkage between antibody or its fragment with liposomal lipid. In another approach antibody is chemically modified to increase its hydrophobicity using a suitable substituent, resulting in higher affinity for bilayers of the liposome.

Current trend is to use the antibody fragments, i.e., fragment antigen-binding (Fab′)/single-chain fragment variable (scFv), instead of using the whole antibody. The main advantage of adapting this strategy is to avoid the risk of inactivation of antibody during surface functionalization to obtain immunoliposomes [102]. The benefits of size reduction of antibody includes avoiding the possibility of initiating an immune response, and formulation of immunoliposomes with a smaller size for efficient delivery of anticancer agents at the requisite site [57]. 

A review has summarized the techniques being used for derivatization of antibody and formation of reactive groups to be conjugated with lipids or preformed stealth liposomes [103]. Several reagents are used for thiolation of antibodies, e.g., 2-Iminothiolane (Traut’s reagent) to form sulfhydryl-group. Antibody molecules have groups, e.g., carboxyl, amine and thiol groups, which can be modified for active targeting. The utilization of sulfhydryl group in coupling of thiolated antibody with the lipid having corresponding reactive group, i.e., MAL, is the key strategy and haw been extensively reported in the literature. Sulfhydryl group is prone to oxidation, which can be avoided by removing the oxygen and adding ethylenediamine tetraacetic-acid [103]. The same strategies have been employed for surface functionalization of liposomes with antibody fragments.

Carbonic anhydrases (CA) are surface antigens (zinc metalloenzymes) found in red blood cells [104,105,106]. Hypoxia is common in many tumors. Hypoxia is accompanied with decrease in extracellular pH (about pH 6.5) in cancer microenvironments [107]. Two carbonic anhydrases (CAs) (IX and XII) are over expressed due to hypoxia (lack of oxygen) in many solid tumors, particularly lung and brain tumor. CA-IX has shown higher activity than CA-XII [108]. Wong et al., (2014) formulated CA-IX targeted immunoliposomes having docetaxel encapsulated. In vitro binding and cellular uptake of the liposomes to A549 cells (CA-IX positiveand CA-IX negative) was studied using fluorescence based flow cytometry, showing higher uptake in CA-IX positive A549 cells [64]. 

In a recently published paper by our research group, liposomes containing triptolide were functionalized with anti-CA-IX antibody, and showed higher efficacy in lung cancer therapy in mice bearing lung cancer [109]. The conjugation of the anti-CA-IX antibody with DSPE-PEG-Mal micelles was performed by treatment with a reducing agent dithiotreitol (DTT) through thiolation-reaction in a PBS buffer (pH 6.6) for 2 h at room temperature. The reduction cleaved the antibody at the hinge and generated cleaved antibodies containing a free-thiol group. Liposomes were prepared by ethanol injection method. CA-IX directed liposomes were formulated by DSPE-PEG-Mal-CAIX micelles incorporation into preformed liposomes. Unreacted antibody and micelles were then removed by Sepharose CL-4B gel filtration.

### 4.2. Surface Modification of Liposomes with Peptides

Liposomes are surface engineered with peptides (a short chain of amino acids) mainly by covalent and non-covalent bonding. Peptides are conjugated with liposomes through covalent bond by a variety of linkages, e.g., MAL linkage bond, peptide bond, sulfanyl bond, disulfide bond and phosphatidylethanolamine-linker bond [110]. Currently, disulfide and thioester linkages have been widely reported in the literature. Amphipathic peptides have been conjugated with the liposomes by non-covalent linkage [111].

Peptides for surface functionalization of nanocarriers, i.e., liposomes, are divided into two categories, i.e., cell-penetrating peptide (CPP) and cell-targeting peptide (CTP), having non-specific and receptor-specific binding and internalization, respectively [112]. Recently, our research group have reported liposomes containing cantharidin and a tumor specific cell-penetrating peptide BR2. BR2-directed liposomes demonstrated enhanced cellular-uptake in hepatocellular carcinoma as compared to non-targeted liposomes [113]. The conjugation of cystein-modified BR2 peptide was done by incubation with a 4-(2-hydroxyethyl)-1-piperazineethanesulfonic acid (HEPES) buffer containing DSPE-PEG-MAL for 48 h at room temperature. The unreacted peptide and l-cysteine were removed by dialysis against distilled water for 48 h. Ethanol injection method was used for formulation of liposomes by incorporation of DSPE-PEG-MAL-BR2 conjugate in the formulation step along with other lipids.

T7 targeted liposomes encapsulating paclitaxel showed enhanced tumor inhibition in ovarian cancer bearing mice as compared to non-targeted liposomes and free drug [114]. Cyclic RGD peptide-modified liposomes containing doxorubicin showed the growth inhibition of human glioma cells (U87MG cell-line). Cyclic RGD has affinity for integrins, over-expressed in many tumors. Integrins also have high specificity for cyclic RGD [94]. PEGylated liposomes containing CPP and acid-sensitive hydrazone bond were prepared. It was found that 4% CPP to lipid ratio showed higher internalization efficiency of the liposomes into targeted compartments [115]. Patra et al., (2016) prepared nano-liposomes containing CPP (polyarginine) and carbon dot for delivery of curcumin across the skin. The presence of carbon dots helped in fluorescence imaging of the skin [116].

### 4.3. Surface Modification of Liposomes with Aptamers

These are short single-stranded RNA or DNA sequences (of oligonucleotides) capable of targeting receptors on the surface of cancer tissues. SELEX (systematic-evolution of ligand by Exponential-enrichment) technology led to the development of aptamers having high affinity for target molecules [117]. Liposomes containing a targeting aptamer ligand linked to the surface of the liposomes and encapsulated anticancer cisplatin were reported. The aptamer possessed affinity for nucleolins (NCL). Aptamer directed liposomes were formulated by addition of the cholesterol-tagged aptamer before the hydration step with other constituents of the liposomal formulation. The aptamer targeted liposomes exhibited enhanced anti-proliferative activity in breast cancer (MCF-7 cells) over expressing NCL as compared to non-targeted drug loaded liposomes and free drug. This study demonstrated the specific targeting of breast cancer cells over-expressing NCL [118]. In a study by Kang et al., aptamer Sgc8 was conjugated to the surface of liposomes and these liposomes were loaded with FITC-dextran. The formulation delivered the encapsulated load to cancer cells, i.e., leukemia cells, by targeting the protein tyrosine-kinase-7 receptor in vitro with high specificity and efficiency [119].

Baek et al., (2014) developed doxorubicin loaded liposomes conjugated with anti-PSMA (prostate-specific membrane antigen) A9 aptamer. The formulations showed an enhanced binding to prostate cancer cells (PSMA positive) along with the reduction in tumor size in prostate epithelial cells (LNCaP) as compared to non-targeted liposomes [120]. Aptamer functionalized liposomes specific for targeting prostate cancer were reported. The aptamer SZT01 recognizes PSMA, over expressed in prostate cancer. The liposomes also encapsulated *N*,*N*,*N*′,*N*′-tetrakis(2-pyridylmethyl)-ethylenediamine (TPEN). This compound chelates zinc, thereby, induces oxidative stress leading to cell death. Liposomes were prepared by film hydration method. Dithiol group was reduced in the aptamer followed by overnight incubation of the aptamer to preformed liposomal suspension. Subsequently, the aptamer directed liposomes were purified. TPEN loaded aptamer targeted liposomes reduced tumor growth in a prostate cancer xenograft model [121]. Targeted delivery of doxotubicin by anti-breast cancer RNA aptamer (TSA14) using PEGylated liposomes has been reported. DSPE-PEG-TSA14 micelles were prepared by incubation of TSA14-NH2 aptamer with DSPE-PEG(2000)-carboxylic acid at room temperature for 2 h. TSA14 directed liposomes were prepared by incubation of DSPE-PEG-TSA14 micelles with preformed liposomes loaded with doxorubicin at 60 °C for 0.5 h. The aptamer directed liposomes were purified by dialysis against 5%-dextrose solution. In vitro studies showed that the liposomal formulations improved cellular uptake and cytotoxicity of doxorubicin in mice bearing breast tumor model as compared to non-targeted liposomes [122].

To overcome multi-drug resistance (MDR) in breast cancer, an aptamer based liposomal formulation was suggested. MDR metastatic breast cancer cells over-express P-gp transporter. This overexpression can be minimized by silencing with siRNA. To enhance the selective delivery of siRNA into breast cancer cells, liposomes using aptamer A6 (having affinity for HER2 receptors on breast cancer cells) as a targeting ligand and loaded with siRNA were prepared. Aptamer A6 was incubated with preformed liposomes having MAL group from PEG-MAL available for conjugation with the aptamer. The incubation resulted in formation of A6 directed liposomes. The study concludes that aptamer directed liposomes could be used for the delivery of siRNA (directed towards P-gp) into the breast cancer cells to overcome chemo-resistance [123].

### 4.4. Surface Modification of Liposomes with Small Molecules

A variety of small molecules have been utilized as targeting ligands, e.g., folate, affibody, carbohydrate, etc., for surface modification of liposomes, for treatment of cancer.

Folate directed liposomes containing imatinib (an inhibitor for platelet-derived growth-factor receptor) have been formulated for cervical tumor therapy. Film hydration method was used for formulation of liposomes and the addition of folate lipid-conjugate was done in the step of lipid film formation. Imatinib was loaded into liposomes by transmembrane pH-gradient. Folate targeted liposomes reduced the IC_50_ value of cervical tumor (HeLa-cells) six folds, i.e., 910 μM to 150 μM [124]. 

A recently published paper has reported HER2 targeting affibody (Z00477)2-Cys conjugated liposomes for treatment of breast cancer, i.e., SK-BR-3 and TUBO cloned-cells. Affibody was reduced and conjugated with DSPE-PEG-MAL micelles by thioether linkage. Cisplatin was dissolved in aqueous phase and loaded into liposomes during the formulation. Liposomes were formulated by ethanol injection method. Cisplatin containing liposomes were surface modified with affibody conjugated micelles by 4 h incubation at 47 °C. The affisomes showed higher cellular uptake and therapeutic efficacy in breast cancer therapy [125].

### 4.5. Surface Modification of Liposomes with Dual-Targeting Ligands

A recent trend is surface functionalization of liposomes with two ligands, i.e., a combination of peptide and antibody targeting ligands in a single formulation. In a study by Kang et al., (2015), chimeric-ligand directed multifunctional liposomes, i.e., folate-linked-peptide-1, and dual ligand, i.e., folate and peptide-1 directed liposomes, were formulated by film hydration method. In the first step, PEGylated liposomes were formulated with terminal MAL group. In the second step, the liposomes were incubated with chimeric-ligand, i.e., folate-linked-peptide 1 or folate and peptide-1 ligand, to formulate chimeric-ligand directed liposomes and dual ligand directed liposomes, respectively. The multifunctional liposomes have been loaded with FITC-dextran and were used against, uterine-cervical cell line (HeLa) and human-keratinocyte cells (HaCaT). In this study, dual ligand directed liposomes showed higher cellular uptake and cytotoxicity in HeLa cells as compared to chimeric-ligand directed liposomes [126].

Recently, Zhang et al., (2017) reported the formation of targeted liposomes containing two peptides (T7 and DA7R are specific peptides for TfR and VEGFR2) and two anticancer drugs (doxorubicin and vincristine) for treatment of glioma (brain tumor). The dual targeting showed improved brain drug delivery and higher therapeutic efficacy [30]. Zong et al., (2014) prepared doxorubicin liposomes containing two peptides (TAT and T7) which showed an improvement in the therapeutic efficacy in treatment of glioma in animals as compared to single ligand doxorubicin liposomes and free doxorubicin [127].

## 5. Stimuli Strategies for Enhanced Delivery of Anticancer Agents at Requisite Location Using Stimuli-Responsive Functionalized Liposomes

Stimuli causes instability to the liposomes leading to the release of entrapped material [128]. Following are the main stimuli being used for enhanced delivery of anticancer agents at requisite site using liposomes (see Table 2).

### 5.1. Temperature Responsive Liposomes

Liposomes are made from thermo-sensitive lipids which are stable at body temperature (37 °C). Thermosensitive liposomes have been used to target tumor cells, which have relatively high temperature compared to normal body. The liposomes prepared from dipalmitoylphosphatidylcholine (DPPC) and cholesterol released about 80% of encapsulated methotrexate within 0.5 h when the temperature was increased from 37 °C to 41 °C. The commercial anticancer liposomal formulation Thermodox^®^ (Celsion, Lawrenceville, NJ, USA) is an example of temperature sensitive liposomes. It contains MSPC (1-myristoyl-2-stearoyl-*sn*-glycero-3-phosphocholine) which has transition temperature of 40 °C [7,129]. 

### 5.2. pH Responsive Liposomes

pH responsive liposomes are stable at pH 7.5; alteration in pH as in tumor tissues, i.e., low pH, causes release of the entrapped payload due to instability of the bilayers [130,131]. The pH can go as low as 5.7 at the tumor site [132]. DODAP or 18:1 DAP is an example of pH sensitive lipid, commonly used to form pH sensitive liposomes [133]. In a study by Zhao et al., (2016), pH responsive liposomes loaded with doxorubicin were formulated for treatment of glioma. DSPE-PEG-H_7_K(R_2_)_2_ was prepared by reaction of DSPE-PEG-NHS with H_7_K(R_2_)_2_. The liposomes were prepared by film hydration method and drug was loaded into liposomes through ammonium-sulfate gradient. Liposomes were surface functionalized with H_7_K(R_2_)_2_, a pH-sensitive peptide. Liposomes showed higher therapeutic efficacy in treatment of glioma (C6-cells) and glioblastoma (U87-MG cells) [134]. Cationic liposomes containing pH sensitive polymer have been suggested as antigen delivery carriers for cancer immunotherapy [135]. In another study, liposomes were loaded with antigenic peptides derived from ovalbumin and pH-sensitive fusogenic polymer in an attempt to establish peptide vaccine-based cancer therapy [136].

### 5.3. Magnetic-Field Responsive Liposomes

These liposomes contain iron-oxide cores (magnetite, Fe_3_O_4_), which become magnetized on the application of external magnetic field [131]. Magnetoliposomes loaded with 5-fluorouracil have been developed. Film hydration method was used to prepare the liposomes. Lipid film of PC solution in chloroform was prepared by evaporation under vacuum, followed by hydration with Fe_3_O_4_ suspension in water. Magnetic field caused the release of the drug due to hyperthermia produced and tumor growth inhibition was observed in human colon cancer cells [137]. Liposomes containing methotrexate and iron-oxide showed more accumulation in the target tissue in a mouse model on the application of an external magnetic field as compared to the same liposomes in the absence of an external magnetic field [138]. 

### 5.4. Ultrasound Responsive Liposomes

Liposomes containing small gas bubbles (produce echo sound upon exposure to ultrasound waves) allow ultrasound imaging. Ultrasound waves can also be used to disrupt liposome structures through ultrasound stimulation, thereby, releasing the drug at the requisite site [139]. Doxorubicin liposomes, possessing CO_2_ bubbles producing thermosensitive system, significantly inhibited the breast tumor growth in MDA-MB-231 tumor-bearing mice compared to simple thermosensitive doxorubicin lipoosmes producing no gas. Thermo-responsive liposomes capable of producing CO_2_ bubbles to disrupt lipid bilayers by hydrating the dried lipid film with citrate buffer (300 mM, pH 4).

An enhancement in the antitumor activity of doxorubicin, owing to the synergism between burst release of drug and hyperthermia induced CO_2_ generation was observed. An ultrasound imaging system was used to monitor hyperthermia-induced CO_2_ generation. In this study, a novel method is used in which the drug is released from liposomes due to hyperthermia leading to CO_2_ generation in liposomes [140].

### 5.5. Other Stimuli Responsive Liposomes

Various other stimuli such as light, redox, enzymes, etc. have been used for improved delivery of anticancer agents at tumor site. Some recent examples of stimuli responsive liposomal delivery systems are described below. A research paper by Li et al. [142] (2017) reported multiple lipid-carrier complexes consisting of a nanostructured lipid-carrier (NLC) within liposomes. The formulation contained a hydrophobic dye IR780 responsive to laser-irradiation in liposomal bilayer and AMD3100 (a drug) in aqueous compartment of liposomes. Accumulation of AMD3100 was observed at cancer site, where it bound to CXCR4-receptors leading to 90% inhibition of metastasis in osteosarcoma (U20S cell-line). Further, in vivo experiments revealed that the system showed complete inhibition of breast cancer (4 T1-luc cell-line) after laser irradiation for three days. Overall, the system showed inhibition of metastasis and higher therapeutic efficacy.

Chi et al. [141] has recently reported redox-responsive, hyaluronic acid functionalized liposomes. Doxorubicin was loaded in liposomes by active loading. Cationic-liposomes were non-covalently coated with hyaluronic acid, a targeting ligand for CD44 receptor. Solvent-injection method was used to prepare cationic liposomes by dissolution of lipids in ethanol, followed by addition of ethanolic solution to the PBS with stirring. HA (negatively charged) solution was coated on the cationic liposomes with 4 h stirring. An ammonium-sulfate gradient was used for loading doxorubicin in the liposomes. The liposomes showed destabilization upon exposure to reducing conditions or low pH. The developed redox responsive liposomes showed a significant increase in cytotoxicity in treatment of osteosarcoma (MG63 cell-line) in vitro and in murine xenograft model.

### 5.6. Liposomes Responsive to Concurrent or Multiple Stimuli

A recent trend is the development of liposomal formulations, responsive simultaneously to a variety of stimuli. Liposomes containing both pH sensitive polymer (2-propyl acrylic acid) and temperature sensitive polymer (NIPAAm) have been prepared using p(NIPAAm-co-PAA) co-polymer. Doxorubicin release was dependent on pH and temperature responses. Film hydration method was used to prepare liposomes and doxorubicin was loaded by pH gradient. MR-guided focused ultrasound was used to heat specific tissues and trigger local drug release. The results showed greater cytotoxicity in breast tumor (MCF-7 cells) by administration of these liposomes [143]. The aim of formulating dual responsive liposomes was to decrease the adverse effects to normal tissues. 

A dual stimuli responsive liposomal system has been developed to overcome the issues, i.e., CPP-degradation in CPP-siRNA conjugate. The liposomal system was responsive to two stimuli, i.e., cellular redox-environment and temperature. The liposomes were formulated by encapsulating the siRNA-CPP inside NGR-peptide directed liposomes. siRNA-CPP conjugate was prepared by conjugation of siRNA with CPP through disulfide-linkage. NGR directed temperature-responsive liposomes were developed by dissolution of DPPC:MSPC:DSPE-PEG(2000)-NGR (87:3:10, weight ratio) in chloroform with subsequent evaporation to form lipid film, followed by hydration of the film with HEPES buffer. The liposomes targeted fibrosarcoma-cells (HT-1080 cell line) and silenced c-myc. The developed liposomal system in combination with hyperthermia, showed three-fold higher therapeutic efficacy and two-fold higher gene-silencing capability in xenograft model established in mice [144].

## 6. Enhanced Delivery of Anticancer Agents at Tumor Site Using Dual Functionalized Liposomes Responsive to Stimuli and Grafted with Targeting Ligands

Development of liposomes responsive to stimuli and surface engineered to target the receptors overexpressed on surface of cancer cells or tumor microenvironment is a promising approach and recent trend to maximize the benefits of cancer therapy. An article has reported formulation of antibody-targeted thermosensitive liposomes. The study reports that thermal and physicochemical properties of traditional thermosensitive-liposomes (TTSL) were maintained after surface functionalization with hCTMO1-antibody. TTSL were prepared by film hydration method. DSPE-PEG-MAL-hCTMO1 micelles were prepared by reaction of thiolated antibody with MAL group. The thermosensitive, targeted liposomal system was formulated through post-insertion technique by incubating DSPE-PEG-MAL-hCTMO1 micelles with preformed TTSL for 1 h at 60 °C. The application of developed liposomal system with heating at 42 °C for 1 h resulted in higher cytotoxicity and cellular uptake as compared to conventional thermosensitive liposomes in breast tumor (MDA-MB-435 cells) overexpressing *MUC1-gene* [145]. 

Table 3 describes recently published data about concurrent use of stimuli and targeting ligand in a single liposomal formulation for efficient delivery of anticancer agents at tumor site. Further, the table also describes the recent techniques used for dual functionalization of liposomes.

## 7. Challenges and Limitations of Functionalized Liposomes as a Carrier for Anticancer Agents

Liposomes like other nanocarriers have challenges and limitations associated with them. Liposomes should be stored in a refrigerator. Liposomes cannot be stored in a freezer because it will lead to formation of ice crystals that may rupture the phospholipid-bilayers in liposomes. Oral administration is more convenient for administration of liposomal formulations, however current liposomal formulations available in the market for cancer therapy are mainly administered by intravenous route [6]. 

In development of an efficient liposomal delivery system, some factors needs to be carefully considered, such as size, charge, shape, ligand density and features of target cell [152]. Liposomes having smaller size have lower ligand–receptor interaction. Shapes such as rod, spheres, cylinders, etc. have demonstrated higher cellular-uptake. Liposomes having positive charge are readily taken up by the cells due to electrostatic-attraction between liposomes and negatively charged cell-membrane. Optimum ligand density on liposomes surface can maximize the liposomal uptake in cancer cells. However, increasing the ligands density beyond optimum concentration will lead to aggregation. Liposomes optimum size for cellular-uptake of liposomes may vary from one cell type to another because of variations in phenotype [153].

Optimal density and length of PEG chains varies in different liposomal formulations and there is a need to establish an optimum range for density and length of PEG chains in different liposomal delivery systems. Longer chains of PEG polymer, e.g., PEG(5000), folds into globular structures such as mushrooms and lack linearity in water. The lack of linearity in PEG structure has led to steric-hindrance in binding of ligand to the receptor. This challenge may be addressed by carefully considering binding affinity of ligand to the receptor [154]. Challenges such as liposomes’ limited ability to penetrate through barriers (higher hydrostatic pressure, disordered vasculature and binding-site barrier) need to be addressed [155]. Functionalization of liposomes with various targeting ligands has resulted in enhanced detection by the immune-system [156]. Targeting capability of the developed liposomal system can be compromised by interaction between serum-protein and ligands [157].

Stability of the liposomal formulation is an important parameter to obtain optimal cytotoxic effect in cancer therapy. The leakage of the drug from liposomes after administration is also an important issue, which needs to be addressed. Charged liposomes have been reported in literature with harmful effects on body. Polyvalent-cationic liposomes have shown pulmonary toxicity in murine model because of reactive-oxygen intermediates [158]. Scaling up the current laboratory based liposomal formulation techniques, e.g., utilization of various surface engineering/functionalization techniques at large scale, is also an important challenge. In this regard, we have to carefully consider the important parameters such as batch reproducibility, stability, entrapment efficiency, sterilization and efficient drug loading strategy. 

The early detection of cancer is still a challenge. The complete recovery of the patients in a short period of time with minimal side effects to normal tissues is the need of the hour. Further research is needed on the recurrence of the cancer and multi drug-resistant cancer. The research may be focused on treating genetic causes of cancer utilizing liposomes as vehicles. Cancer cells observe enhanced-permeability and retention (EPR) effect; the research should be directed to deliver higher amounts of drug at the required sites so that patient gets treatment in a short period of time [36,44]. Danvier and Preat (2015) also suggested improving EPR effect for the delivery of anticancer drugs. The strategies suggested included normalization of tumor vasculature and use of stimuli to increase tumor cell permeability [159].

Targeted delivery of anticancer agents to brain using a nanocarrier (liposomes) is also an important challenge, required to be addressed. We have to address the issue of designing a liposomal delivery system capable of overcoming the dual challenge of penetrating two barriers, i.e., blood–brain barrier (BBB) and tumoral barrier. An efficient strategy for the treatment of brain tumor (glioma) using targeted-liposomes loaded with anticancer agents needs to be developed [160,161].

The cost of commercial anticancer liposomal formulations are very high at the moment [6]. Expensive raw material is the primary reason for high cost of liposomal formulations available in market. Therefore, liposomal anticancer therapy is an economic burden on the patient and health care system. More research is needed, focusing on the large scale formulation methods of liposomes. In this connection, improvement in the existing or development of the new methods may be helpful. 

## 8. Conclusions and Future Perspective

**Figure 4:**
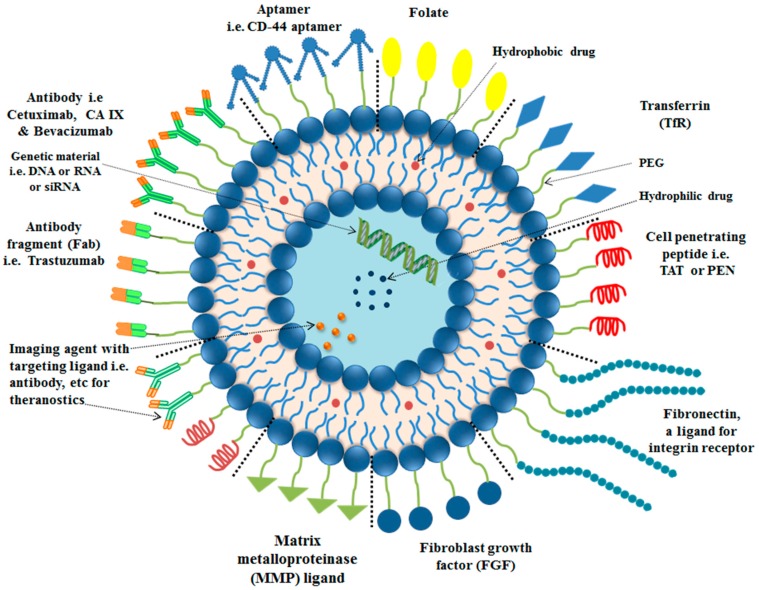
Surface functionalized liposome (summary) with various targeting ligands for enhanced delivery of payload at tumor site.

Tumor biology is an important aspect to be considered in designing a liposomal delivery system. An insight into the exact mechanism for a nanocarrier, i.e., liposomes, to reach the tumor site and exert therapeutic response can play a vital role in addressing the current challenges of tumor therapy. Variation of physico-chemical properties results in dramatic-variations in secondary properties of functionalized nanocarriers, i.e., liposomes [162]. Special attention should be given to the regulatory approval and quality control of liposomes. Functionalization of liposomes with targeting ligands should be done without inactivation of the targeting ligands, using a suitable surface functionalization approach. Optimization of ligand’s density on surface of functionalized liposomes, with minimum variation in the circulation time is an important parameter to be considered. Application of the strategies described in this review article, involving a variety of surface engineering techniques to design and formulate a liposome based delivery system having multiple functionalities in a single formulation, e.g., receptor-specific targeting and stimuli sensitivity, can play a vital role in addressing the current challenges, e.g., multi drug-resistance (MDR) (see Figure 4). To develop a liposomal formulation with high therapeutic efficacy and minimum side effects, cooperation among all related experts involved in the development of liposome technology such as pharmacists, chemists, biologists, pharmacologists, toxicologists, regulatory affair experts, etc. is necessary.

Liposomal formulations have been successfully used in the treatment of solid tumor. Several liposome formulations are commercially available in the market, after regulatory approval from FDA. Tumor therapy in clinics is primarily based on these commercial non-targeted liposomal formulations, exploiting the EPR effect. However, an efficient strategy for tumor therapy is utilization of the targeted liposomal formulations, minimizing the off-target effects. 

In the future, liposomes will play a vital role in personalized-medicine for individual patients by reducing cost and duration of therapy. Multifunctional liposomes having certain features, e.g., sustained release, targeted delivery, triggered release and synergistic functionalities using a variety of surface functionalization and modification approaches, will play a vital role in tumor therapy.

## Figures and Tables

**Table 1:** Surface engineered liposomes functionalized with targeting ligands for solid tumor therapy.

Targeting Ligand	Anticancer Agent	Targeting Site	Surface Engineering Technique Used	Drug Loading	Tumor Treated	Reference
Fab′ fragments of mAb C225 (cetuximab)	Doxorubicin	EGFR	FabV fragments were covalently linked to the maleimide (MAL) group of DSPE-PEG-MAL. Incorporation of DSPE-PEG-MAL-FabV into preformed liposomes by coincubation at 55 °C for 30 min.	Passive loading	Breast cancer	[61]
Anti EGFR antibody	Small interfering RNA (siRNA)	EGFR	Thiolated antibody were conjugated to the MAL group at distal end of DSPE-PEG-MAL chains of preformed liposomes (0.2:1, Ab and MAL molar ratio) by over-night incubation at 4 °C with mild shaking.	Active loading	Lung cancer	[62]
Anti HER2 antibody i.e., fragments of trastuzumab-mAb	Doxorubicin	HER2	Covalent conjugation of Fab or scFv to drug loaded liposomes by thioether linkage of free thiol of Ab-fragment and MAL group or alternatively thiol group was conjugated to terminal MAL group present on surface of liposomes.	Active loading by ammonium-sulfate gradient	Breast cancer	[63]
CA-IX Antibody	Docetaxel	CA-IX	Antibody was reduced by reaction with DTT. Conjugation of DSPE-PEG-MAL micelles with the reduced antibody was done by incubation at room temperature for 24 h. DSPE-PEG-MAL-Ab micelles were incubated with preformed liposomes at 60 °C for 2 h (post insertion technique) to formulate CA-IX directed liposomes.	Passive loading	Lung cancer	[64]
Anti-MT1-MMP Fab	Doxorubicin	MT1-MMP	Fab fragments were conjugated to the MAL moiety in preformed liposomes at a molar ratio of 1:3 respectively in an incubation at 4 °C for 20 h.	Active loading by ammonium-sulfate gradient.	Fibrosarcoma, i.e., HT-1080 cancer cells	[65]
Trastuzumab (Anti-HER2)	Paclitaxel and Rapamycin	HER2	Thiolated antibody was conjugated to the MAL group of DSPE-PEG-MAL in preformed liposomes by overnight incubation and purification with CL-4B column.	Passive loading	HER2 (+) breast cancer, RAP acts synergistically	[66]
Anti VEGFR2	Doxorubicin	VEGFR2	Fab′-Mal-PEG-DSPE was incorporated into the preformed liposomes by coincubation at 55 °C for 30 min. This step was followed by purification with gel filtration.	Passive loading	Colon Cancer	[67]
**Liposomes for Intracellular organelle targeting**						
Triphenyl-phosphonium (tpp)	Paclitaxel	Mitochondrial targeting; higher accumulation of payload in mitochondria	CTPP was incubated for 2 h with triethylamine, EDCI and NHS in choroform at 25 °C with stirring. NH2-PEG-PE prepared was added to the chloroform solution, stirred overnight and chloroform was evaporated. Purification of reaction mixture was done by dialysis with water. Freeze drying was done to get the purified TPP-PEG-PE polymer. The polymer was used in preparation of TPP directed liposomes by film hydration method.	Passive loading	Breast Cancer	[68]
Rhodamine-123	Paclitaxel	Mitochondria	DOPE, NPC-PEG were dissolved in chloroform in presence of triethylamine with overnight stirring. Chloroform was removed from reaction mixture and then reaction mixture was freeze dried. The reaction mixture was hydrated with water to get pNP-PEG-DOPE micelles. Rh123-PEG3400-DOPE was synthesized by dissolution of Rh123 (a fluorescent probe) in methanol and triethylamine solution. Addition of the solution was done to the pNP-PEG-DOPE in chloroform, followed by incubation at 25 °C with stirring for 4 h. Liposomes were prepared using the above polymer by film hydration method.	Passive loading	Cervical cancer	[69]
KLA-peptide having terminal cysteine	Paclitaxel	Mitochondrial targeting of A549 cancer cells and pH responsive liposomal system	In first step, DSPE-KLA was obtained by conjugation of DSPE-Mal with KLA-Cys peptide in chloroform and methanol mixture. Further, DSPE-KLA-DMA was synthesized by dissolution of DSPE-KLA in dichloromethane containing N, *N*-Diisopropylethylamine (DIPEA) and 2,3-dimethylmaleic-anhydride (DMA) and agitation in ice and water bath for 24 h. Film hydration method was used for preparation of DSPE-KLA-DMA (DKD) directed liposomes.	Passive loading	Lung cancer (A549 cell-line)	[70]

**Table 2:** Stimuli-responsive functionalized liposomes for enhanced delivery of anticancer agents at tumor site.

Stimuli Used	Anticancer Agent	Targeting Site	Targeting Ligand	Lipids Used	Technique Used for Functionalization of Liposomes	Liposome Formulation Method	Drug Loading	Tumor Treated	Reference
Temperature	Doxorubicin	-	-	DPPC:MSPC:DSPE-PEG(2000) (86.5:9.7:3.8, mol %)	Liposomes were formulated by film hydration method by formation of lipid film, followed by hydration with citrate-buffer at 50 °C for 10 min.	Film hydration method	pH gradient	Ovarian Cancer	[129]
pH	Doxorubicin	-	H_7_K(R_2_)_2_, a pH-sensitive peptide	DOPE, DSPE-PEG-H_7_K(R_2_)_2_	DSPE-PEG-H_7_K(R_2_)_2_ was prepared by reaction of DSPE-PEG-NHS with H_7_K(R_2_)_2_. H_7_K(R_2_)_2_-targeted liposomes were prepared by film hydration method.	Film hydration method	Ammonium-sulfate gradient	Glioma (C6-cells) and glioblastoma (U87-MG cells)	[134]
Magnetic field	5-Fluorouracil	-	-	Phosphatidylcholine (PC)	Film hydration method was used to prepare magnetoliposomes. Lipid film of PC solution in chloroform was prepared by evaporation under vacuum, followed by hydration with Fe_3_O_4_ suspension in water.	Film hydration method	Passive loading	Human colon carcinoma T-84 cell lines	[137]
Temperature (for drug release) and ultrasound waves (for drug release monitoring)	Doxorubicin	-	-	DPPC:MSPC:DSPE-mPEG(2000) (21.6:2.6:1.0, molar ratio)	Thermo-responsive liposomes (capable of producing CO_2_ bubbles on hyperthermia) by hydrating the dried lipid film with citrate buffer (300 mM, pH 4).	Film hydration method	Ammonium-sulfate gradient	Breast tumor (MDA-MB-23)	[140]
Redox	Doxorubicin	CD44 receptor	Hyaluronic acid	SPC, DOPE, DOTAP, Chol-SS-mPEG or Chol-mPEG	Solvent-injection method was used to prepare cationic liposomes by dissolution of lipids in ethanol, followed by addition of ethanolic solution to the PBS with stirring. HA (negatively charged) solution was coated on the cationic liposomes with 4 h stirring. An ammonium-sulfate gradient was used for loading doxorubicin in the liposomes.	Solvent injection method	Ammonium-sulfate gradient	Osteosarcoma (MG63 cell-line)	[141]
Laser irradiation	AMD3100 and IR780 (a dye)	CXCR4-receptors	-	Soybean-phosphatidylcholine (SPC)	Multiple lipid-carrier complex consisting of a nanostructured lipid-carrier (NLC) within liposomes have been formulated. IR780-loaded NLC have been formulated by film-dispersion method. The dried lipid film for liposome formulation was hydrated with water containing IR780-loaded NLC and AMD3100.	Preparation of NLC by film-dispersion method and formulation of liposomes by film hydration method	Passive loading	Osteosarcoma (U20S cell-line) and Breast cancer (4 T1-luc cell-line)	[142]
**Liposomes responsive to multiple stimuli**									
pH and Temperature	Doxorubicin	-	-	pH-sensitive polymer (2-PAA) and temperature sensitive-polymer (NIPAAm)	Film hydration method was used to prepare liposomes and doxorubicin was loaded by pH gradient. MR-guided focused ultrasound was used to heat specific tissues and trigger local drug release.	Film Hydration Method	pH gradient	Breast tumor (MCF-7 cells)	[143]
Cellular redox-environment and temperature	CPP-siRNA conjugate.	c-myc gene	NGR-Peptide	DPPC:MSPC:DSPE-PEG(2000)-NGR (87:3:10 weight ratio)	siRNA-CPP conjugate was prepared by conjugation of siRNA with CPP through disulfide-linkage. NGR directed temperature-responsive liposomes were developed by dissolution of DPPC:MSPC: DSPE-PEG(2000)-NGR (87:3:10, weight ratio) in chloroform with subsequent evaporation to form lipid film and then the film was hydrated with HEPES buffer.	Film hydration method	Passive loading	Fibrosarcoma-cells (HT-1080 cell line)	[144]

**Table 3:** Dual functionalized liposomes responsive to stimuli and grafted with targeting ligands.

Targeting Ligand	Stimuli Used	Anticancer Agent	Targeting Site	Lipids Used	Techniques Used in Functionalization of Liposomes with Stimuli and Targeting Ligand	Liposome Formulation Method	Drug Loading	Tumor Treated	Reference
hCTMO1 antibody	Temperature	Doxorubicin	*MUC1-gene*	DPPC, HSPC, DSPE-PEG(2000)	DSPE-PEG-MAL-hCTMO1 micelles were prepared by reaction of thiolated antibody with MAL group. Thermosensitive targeted-liposomes were formulated using postinsertion technique by incubation of thermosensitive liposomes with DSPE-PEG-MAL-hCTMO1 micelles for 1 h at 60 °C.	Film hydration method	Ammonium-sulfate gradient	Breast Cancer	[145]
AS1411 aptamer	Temperature	Gd-DTPA	Nucleolin Receptor	DPPC, MSPC, DSPE-PEG(2000)-COOH	Thermosensitive liposomes (TSL) were conjugated with the AS1411-aptamer by utilizing terminal –COOH group present on formulated liposomes. Addition of TSL was carried out with stirring into MES buffer at pH 6 containing sulfo-NHS and EDC. Subsequently, AS1411 (aptamer) was added and stirring was done for 6 h.	Film hydration method	Passive loading	Breast cancer	[146]
HER-2 antibody	Near-infrared light	Doxorubicin and hollow-gold nanospheres (HAuNS)	HER2	HSPC, DPPC, DSPE-PEG(2000)NH-MAL	DSPE-PEG(2000)-NH-MAL, HSPC, DPPC and cholesterol were dissolved in chloroform. A solution of OMP (Octa-decyl-3-mercaptopionate) modified HAuNS in dichlormethane was added into the above lipid mixture dissolved in chloroform. Subsequently, a dry lipid film was formed and hydrated. Finally, HER2 targeted liposomes were formulated by HER-2 antibody overnight incubation (at 4 °C) of preformed liposomes with HER2-antibody.	Film hydration method	Ammonium-sulfate gradient	Ovarian cancer (SKOV3 cells), Breast cancer (BT474 cells)	[147]
Fab′fragment of ErbB2 antibody	pH	Doxorubicin	HER2 Receptor	GGLG, PEG-DSPE, Fab′-MAL-PEG-Glu2C18	pH responsive liposomes were formulated by dissolution of lipids in t-butyl alcohol at a temperature of 60 °C. This step was followed by freeze drying, yielding a mixture of dried lipid powder subsequently hydrated with 30 mM citrate-solution for 2 h. Immunoliposomes were formulated by a covalent (thioether) linkage between thiol group of Fab′ and terminal MAL group present on preformed liposomes. Preformed pH-responsive liposomes were incubated with the Fab′ with stirring at room temperature for 6 h.	Lipid powder mixture preparation by lyophilization and hydration with PBS at 60 °C	Active loading	Breast Cancer (HCC1954 cell-line)	[148]
RGD-peptide	pH	Docetaxel	αVβ3 integrin receptor	PE, linoleic acid (LA), RGD-PEG-LA	Cholesterol, phospahtidyl-ethanolamine (PE), Docetaxel, linoleic acid (LA) and RGD-PEG-LA were dissolved in chloroform and a thin lipid film was formed by evaporation under vacuum using a rotary-evaporator. Subsequently, hydration of the lipid film was done with PBS (pH 7.4).	Film hydration method	Passive loading	Breast tumor (MCF-7 cells)	[149]
Folate	Temperature	Doxorubicin	Folate receptor	DPPC, DSPE-PEG(2000), DSPE-PEG-Folate	DSPE-PEG-FA was prepared by the carbodiimide mediated conjugation of folic acid with DSPE-PEG-NH2. Folate directed thermosensitive liposomes were formulated by film hydration method. A thin lipid-film was prepared after evaporation of lipids, i.e., DPPC:DSPE-PEG(2000):DSPE-PEG-Folate, and cholesterol dissolved in a chloroform:methanol mixture in a round-bottom flask. Subsequently, lipid-film was hydrated and extruded. Liposomes were loaded with doxorubicin using modified ammonium-sulfate gradient.	Film hydration method	Modified ammonium-sulfate gradient	Cervical cancer (HeLa cells) and Cervical-adenocarcinoma (KB cells)	[150]
Anti-EphA10 antibody	pH	MDR1-siRNA	EphA10receptor	PC, DOPE, DOTAP, Chol-SIB-PEG	Lipids, cholesterol and Chol-SIB-PEG were dissolved in dochloromethane and evaporated under vacuum to form a thin film. The film was hydrated with water, sonicated for four minutes and passed through 0.2 μm membrane to formulate Chol-SIB-PEG-modified liposomes (PSL). Surface modification of PSL with anti-EphA10 antibody was done by addition of sulfo-NHS and EDCI solution in PBS (pH 7.4) to the liposomal suspension with stirring for 2 h. This step was followed by addition of anti-EphA10 antibody to the liposomal suspension and overnight incubation at 4 °C.	Modified film-dispersion hydration method	Active loading	Multi-drug resistant breast tumor (MCF7/ADR cells)	[151]
